# Local infectious disease experience influences vaccine refusal rates: a natural experiment

**DOI:** 10.1098/rspb.2022.1986

**Published:** 2023-02-08

**Authors:** Konstantinos Angelopoulos, Gillian Stewart, Rebecca Mancy

**Affiliations:** ^1^ Economics, Adam Smith Business School, University of Glasgow, University Avenue, Glasgow G12 8QQ, UK; ^2^ School of Biodiversity, One Health and Veterinary Medicine, University of Glasgow, University Avenue, Glasgow G12 8QQ, UK; ^3^ CESifo GmbH, Poschingerstrasse 5, 81679 Munich, Germany; ^4^ MRC/CSO Social and Public Health Sciences Unit, University of Glasgow, Clarice Pears Building, 90 Byres Road, Glasgow, G12 8TB

**Keywords:** vaccine refusal, local disease experience, natural experiment, surveillance data, smallpox

## Abstract

Vaccination has been critical to the decline in infectious disease prevalence in recent centuries. Nonetheless, vaccine refusal has increased in recent years, with complacency associated with reductions in disease prevalence highlighted as an important contributor. We exploit a natural experiment in Glasgow at the beginning of the twentieth century to investigate whether prior local experience of an infectious disease matters for vaccination decisions. Our study is based on smallpox surveillance data and administrative records of parental refusal to vaccinate their infants. We analyse variation between administrative units of Glasgow in cases and deaths from smallpox during two epidemics over the period 1900–1904, and vaccine refusal following its legalization in Scotland in 1907 after a long period of compulsory vaccination. We find that lower local disease incidence and mortality during the epidemics were associated with higher rates of subsequent vaccine refusal. This finding indicates that complacency influenced vaccination decisions in periods of higher infectious disease risk, responding to local prior experience of the relevant disease, and has not emerged solely in the context of the generally low levels of infectious disease risk of recent decades. These results suggest that vaccine delivery strategies may benefit from information on local variation in incidence.

## 1. Introduction and background

Despite the critical importance of vaccination to public health (e.g. [1]) and the excellent safety record of vaccines [2], recent years have witnessed an epidemiologically significant increase in vaccine refusal. This rise in vaccine refusal has contributed to outbreaks of previously rare or even locally eliminated diseases such as pertussis, rubella and measles [1,3–7], as well as to difficulties in controlling new diseases such as COVID-19 [8].

Vaccine refusal is the outcome of multiple contributing factors (e.g. [9]). For example, in the ‘3 Cs’ model of vaccine hesitancy, possible determinants are organized into three types: convenience, confidence and complacency [10]. In this framework, *convenience* focuses on factors such as availability, accessibility and affordability, *confidence* relates to trust in the effectiveness and safety of vaccines and how and why they are delivered, and *complacency* captures the idea that ‘perceived risks of vaccine-preventable diseases are low and vaccination is not deemed a necessary preventive action’ [10, p. 4162]. Confidence thus captures perceptions of both the effectiveness of vaccines and risks associated with vaccination while complacency relates to the evaluation of benefits from vaccination in terms of reducing disease risk. Vaccine uptake or refusal is determined by balancing perceived risks associated with the vaccine and the disease it targets (e.g. [1]), an evaluation that takes place under constraints related to access to vaccination and treatment (e.g. [11]). Analysis from earlier periods suggests similar processes acting as far back as the eighteenth century, with parents who avoided the inoculation of their children against smallpox often attempting to balance these competing risks [12]. The evaluations leading to vaccine uptake or refusal are also affected by values, attitudes and social influences that are typically related to social class and other socioeconomic characteristics (SC) and that can affect an individual's perception of risks [7,11,13–16]. Research has also shown that rates of vaccine refusal vary spatially at different scales—internationally [17], within the same country [1] and even within smaller geographical areas such as states [18–22]—suggesting that the relative strength of different determinants also varies spatially.

Here, we examine the contribution to vaccine refusal of prior local disease experience, using data on variation in vaccine refusal and its potential determinants from small areas within Glasgow at the beginning of the twentieth century. Prior disease experience should affect vaccine refusal via complacency: experience of high levels of disease can serve as a reminder of risks of the disease and thus contribute to reducing vaccine refusal, whereas low levels might increase complacency. The hypothesis that complacency contributes to vaccine refusal is consistent with the recent increases in refusal which have arisen during an extended period of low levels of life-threatening infectious disease; indeed, these recent increases in vaccine refusal have been linked to complacency in research and expert evaluations [9,10,16,23–26]. We ask whether local disease experience contributes to explaining subsequent vaccine refusal during a period characterized by rapid declines in infectious disease risk relative to previous centuries, but nevertheless much higher incidence and mortality risk than during recent decades. A negative relationship between disease experience and vaccine refusal in our analysis would be supportive of the complacency hypothesis, indicating that complacency also influenced vaccination decisions in periods of higher infectious disease risk in response to local prior experience of the relevant disease and has not emerged solely in the context of the generally low levels of infectious disease risk of recent decades.

## 2. Methods

We exploit the natural experiment generated by two smallpox epidemics between 1900 and 1904 in Glasgow and the Vaccination (Scotland) Act 1907 [27] that allowed vaccine refusal, following the period 1863 to 1907 during which smallpox vaccination of infants was compulsory. To study the relationship between smallpox experience and vaccine refusal, we use surveillance data from the constituent administrative units of the city of Glasgow and exploit variation in smallpox incidence and mortality between these units from the two epidemics during the period 1900–1904 and in parental refusal to vaccinate infants between 1907 and 1913 (until the First World War). The timing of events ensures that vaccine refusal after 1907 could not have affected smallpox incidence in 1900–1904. During the period we study, smallpox was the only infectious disease for which a vaccine was readily available, thus there cannot be carry-over effects from refusal of vaccines against other diseases.

There are high-quality small-area records of both smallpox incidence and vaccination outcomes for the period of our study. Throughout the period under consideration, Glasgow's Medical Officer of Health was charged with producing annual reports on the health of the inhabitants under his jurisdiction, including information collected by the district registrars, who, in addition to information on ‘vital events’, collected information on vaccination for each registration district. Our dataset is based on the administrative records created by the Medical Officer of Health (presented by municipal ward) and the district registrars (presented by registration district). Clinically, smallpox was readily identifiable by trained personnel and was also well recognized among the general population [24]. Furthermore, it was a notifiable disease [28] so all cases had to be notified by law and prosecutions were made for non-notification, while known contacts were placed for observation in reception houses [29,30]. Vaccination was a legal requirement and vaccine refusal, when permitted after 1907, was also governed by a legal process. Therefore, the records we use should be highly accurate.

### (a) Context of the natural experiment

In this section, we briefly explain the historical context of the natural experiment that generates the data we use in our analysis. Further detail of the historical context is provided in Section A of the electronic supplementary material and additional information on the variables we construct is provided in the Data section below.

Historically a major killer, smallpox mortality reduced substantially over the nineteenth century. A first important reduction was observed as initially voluntary vaccination became more common and as a result of other public health measures such as the isolation of patients and contacts; following the introduction of compulsory vaccination, there was a further reduction in mortality (see [31] for an analysis of London, and [32] for the earlier period in England). In Glasgow, there were 2197 smallpox deaths during the decade leading up to the introduction of compulsory vaccination in Scotland (1855–1864); by contrast, there were 967 in the subsequent 10-year period (1865–1874) and only 122 over the 25-year period 1875–1899 [30] (electronic supplementary material, Section A).

Smallpox vaccination of infants was made mandatory in Scotland in 1863 and was compulsory until 1907. According to the Vaccination (Scotland) Act of 1863, children were required to be vaccinated by the age of six months [33, p. 3]. In particular, the parents had the responsibility to arrange vaccination and provide the district registrar with the appropriate certificate. Vaccinations were carried out by a doctor and incurred a fee; the exception was that for very poor families, provision was made for free vaccination [34], although this represented only a very small proportion of vaccinations in Glasgow in the early twentieth century (electronic supplementary material, Section A). Data between 1899 and 1906 reveal that most infants were vaccinated (about 94% of infants who did not die before vaccination) (electronic supplementary material, Section A). Indeed, only around 4% of infants who did not die before vaccination in the years prior to 1906 were recorded under the category ‘Removed from District before vaccination, or otherwise unaccounted for’. The remainder was made up of infants recorded in the categories ‘Vaccination postponed’ (used for medical reasons) or ‘Insusceptible to vaccination’.

**Figure 1.**
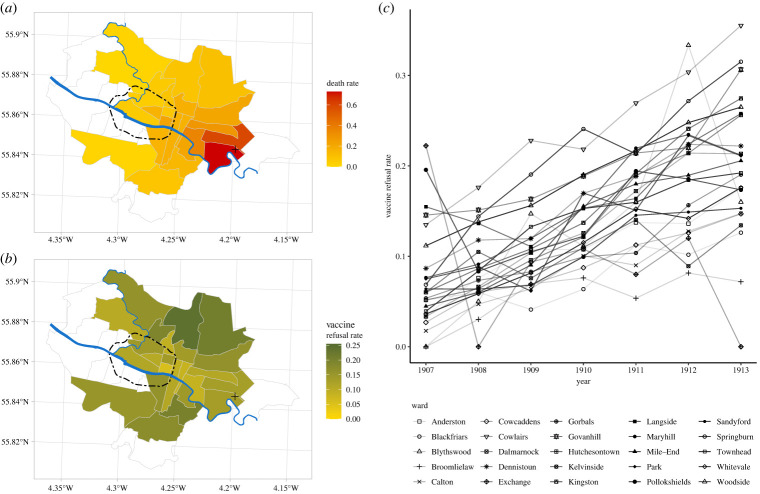
Death rates and vaccine refusal rates across wards of Glasgow using maps of Glasgow [36] and time series of vaccine refusal rates by municipal ward. (*a*) Map of the crude death rate per 1000 population by ward during the two smallpox epidemics of 1900 and 1903, with the Belvidere Smallpox Hospital indicated with a black cross, the River Clyde and River Kelvin shown in blue and the route of the Glasgow Subway indicated with a dashed black line (wards in white are not part of our analysis because they joined the city after the 1900 and 1903 epidemics). (*b*) Map of average vaccine refusal rates (number of infants whose parents registered conscientious objection to smallpox vaccination as a share of children born in the ward) over the period 1907–1913. (*c*) Time series of vaccine refusal rates by ward in Glasgow, 1907–1913.

Glasgow experienced two smallpox epidemics during the period 1900–1904, with the data we have compiled showing that case numbers differed by nearly two orders of magnitude between municipal wards and that the number of deaths per ward ranged from 0 to 69. The first, and larger, of the smallpox epidemics took place during 1900–1901 [35] while the second occurred during 1903–1904 [30]. In figure 1*a*, we show a map of the distribution of death rates across wards (see electronic supplementary material, Section A for the construction of maps and for statistics for cases, deaths, case rates and death rates, showing these different measures demonstrated very similar spatial patterns, further implying that the case fatality ratio across wards was also very similar). During the two epidemics, a similar spatial pattern was observed, in that higher numbers of cases and deaths were observed in the eastern wards which generally had poorer socioeconomic conditions and which were also closer to the Belvidere Smallpox Hospital, the designated hospital for smallpox treatment in the city. Although almost all identified cases were transferred to hospital (all but 10 of the 1759 cases during the 1900 epidemic, [35]), cases and deaths in the MOH reports were recorded according to location of residence. Given the high vaccination rates of infants against smallpox, the majority of deaths during the epidemics were among adults (electronic supplementary material, Section A).

The press and the public viewed the two epidemics in Glasgow as alarming, and awareness was widespread. Indeed, the 1900 epidemic was reported in the *Glasgow Daily Mail* on 13 March 1901 as the most important outbreak in a generation. The epidemics were also frequently reported in both the Glasgow press and outside of the city, such as in regular reports during the first quarter of 1901 in the *London Daily News* and the *Globe*. Awareness was also raised through a concerted effort to encourage revaccination in Glasgow [35], instigated because the effectiveness of the vaccine waned after around 10–15 years [37]. Indeed, around 400 000 people were revaccinated by 1901 [35,38,39], with revaccination proving 100% effective against symptomatic infection and death during the contemporaneous epidemic [35,39]. One possible implication of the revaccination programme is that it may have decreased the incentives to vaccinate infants by generating the expectation that vaccination should be offered during epidemics. On the other hand, the fact that those who died were mostly individuals who had not recently been vaccinated should have positively influenced perceptions of the value of the vaccine. In general, the popularity of the revaccination programme suggests higher demand for vaccines during a period of increased risk, consistent with the complacency hypothesis. Unfortunately, spatial variation in revaccination rates cannot be analysed quantitatively because available information suggests that any effects from ongoing transmission were very localized. For example, The Medical Officer for Health [35, p. 22] commented that people took up revaccination in the tenements where there had been an outbreak but that in the rest of the ‘infected districts’ there was ‘complete apathy’ to revaccination. Analysis of the contemporaneous relationship would also be complicated by possible reverse causality effects because higher rates of revaccination should also translate into lower incidence.

In Scotland in the period following the 1863 Vaccination Act [34], there was not a strong anti-vaccination movement, at least in comparison to the situation in England [34]. For example, the first formal Scottish Anti-Vaccination League was founded in 1896 [40], over two decades later than the corresponding league in England, founded in 1874 [41, p. 39]. From 1867 onwards, standardized lymph production and distribution meant that vaccines in Scotland were generally considered safe [34]. Furthermore, despite the administrative burden of recording vaccination outcomes, medical practitioners in Scotland did not complain to the same extent as those in England [34]. Opposition in England led to the 1898 Vaccination Act [42], allowing for the provision of ‘conscientious objection’ to vaccination (COV) in England; in 1907, further legislation was passed in England to simplify the process [43]. In Scotland, however, it was not until 1907 that the legislation changed to permit COV [27].

The Vaccination (Scotland) Act 1907 [27] allowed parents in Scotland to register COV of their children against smallpox, setting out the process accordingly. A simple form was provided, with blanks for the names of the parent and child and spaces for the signature of the parent and a magistrate or a solicitor before whom the declaration was made (see electronic supplementary material, Section A). Given that literacy rates were very high in Scotland following the Education (Scotland) Act of 1872 [44], with illiteracy having been ‘virtually eliminated’ by the end of the nineteenth century [44, p. 219], the form should have been straightforward to complete. The declaration had to be returned by the parent to the registrar of the district of birth within 7 days [27], either by hand or by post [45, p. 176]. Overall, both the process of vaccination of infants and that of submitting a COV implied some financial and/or time or other administrative costs.

Vaccination refusal rates varied substantially between administrative units after 1907 (figure 1*a,c*; see electronic supplementary material, Section A for the equivalent of figure 1*b* for each year). For example, in 1907, the proportion of children whose parents submitted a conscientious objection form as a share of the children born in the ward ranged from 0 to over 20%. Vaccine refusal rates subsequently increased, but between-ward variation was maintained over time. Data on the full range of vaccination outcomes show that in addition to the sustained increase in vaccine refusal starting in 1907, there was a one-off increase in 1907–1908 in the residual vaccination outcome that recorded children who were ‘Removed from District before vaccination, or otherwise unaccounted for’ (electronic supplementary material, Section A).

### (b) Statistical model

Our aim is to estimate the effect of local smallpox experience from epidemics during the period 1900–1904 on vaccine refusal following the introduction of legislation in 1907 that gave parents the right to declare conscientious objection to smallpox vaccination of their children. Our identification strategy, based on the information available from municipal records, is to interpret the variation in disease experience among administrative units as a conditionally randomized natural experiment, where the conditioning is based on observable SC recorded in the municipal records and census reports. Different administrative units were subjected to differential smallpox experience (‘treatment’, in the language of natural experiments) as a result of the two epidemics between 1900 and 1904, taking the form of different levels (or ‘doses’) of disease experience. The timing of events precludes feedback effects from vaccination refusal to smallpox experience. However, SC may confound the effect of prior smallpox experience on future vaccination refusal to the extent that they relate to both. First, SC might have influenced vaccine refusal directly (and irrespective of prior experience), for example by influencing confidence in vaccine safety and efficacy or expectations about exposure to future epidemics, or by influencing the evaluation of relative costs of vaccination versus vaccine refusal. Second, SC are likely to have contributed to disease transmission and thus the strength of prior smallpox experience during the epidemics. There was a similar spatial pattern in disease experience in the two consecutive epidemics, with higher numbers of cases and deaths in administrative units closer to the smallpox hospital. Because these administrative units were also characterized by lower socioeconomic conditions, this pattern may be (partially) driven by underlying socioeconomic conditions that made these administrative units generally more vulnerable to infectious disease than others. Given the above considerations, we estimate the effect of prior disease experience on future vaccination refusal conditional on SC that we include in our model specification.

We first estimate a linear specification. Denoting by *y* the random variable that captures vaccine refusal rates after 1907, the baseline statistical model is given by

2.1 $$y_{it}\;=\;\beta_{1}\;+\;\beta_{2}h_{i}\;+\;\beta_{3}T_{t}\;+\;\beta_{4}h_{i}\;\times \;T_{t}\;+Z\gamma \;+\;u_{it},$$

for administrative unit i=1,…, N and year t=1,…, 7 (corresponding to 1907–1913). In this specification, $y_{it}$ is a measure of vaccine refusal in administrative unit *i* and year *t*; $h_{i}$ is the measure of smallpox experience during the 1900–1904 epidemics; $T_{t}$ is a time trend capturing changes over time that are common to all administrative units; Z is a matrix containing the set of variables relating to SC of the administrative units, associated with the coefficient vector γ; and the constant term $\beta_{1}$ captures common factors, including shared knowledge about the 1900–1904 epidemics. Finally, $u_{it}$ is an error term assumed to be independently distributed between administrative units; we do not assume the error term is independently distributed within administrative units because of possible persistence of unobserved unit-level factors that may have mattered for vaccine refusal. For example, these may include random factors that differed between administrative units but that persisted within administrative units post-1907 and that may have affected people's beliefs, such as pro- or anti-vaccination personalities who were influential in community networks such as churches, schools or sports clubs. Similar specifications to (2.1) have commonly been applied in programme evaluation [46] and have also been used to estimate the effects of historical experience on future outcomes [47].

The interaction term $h_{i}\;\times \;T_{t}$ is included in (2.1) to allow the effect of prior disease experience on vaccine refusal to change over time. It is plausible that the effect of past experiences on decision making fades over time if higher weight is placed on more recent events. In the context we model, a declining effect could also result from an increase over time in the proportion of residents in a given administrative unit who had relocated from elsewhere. At the time, most households lived in rented accommodation and moved house fairly frequently, although commentary in the Medical Officer of Health reports and other documents [48,49] suggests that in Glasgow this was usually within the same neighbourhood or to one of similar characteristics (for a quantitative analysis of house moves in Belfast for the same time period, see [50]). It is nonetheless reasonable to expect that for a given administrative unit, the proportion of residents for whom the 1900–1904 smallpox epidemic experience of that unit differs from their own increased between 1907 and 1913, such that the *effective* experience of the smallpox epidemics actually declined. The specification including the interaction term thus allows us to capture this (time-varying) effective smallpox experience for administrative unit *i* in year *t*.

We estimate the model parameters in (2.1) using least-squares and compute clustered standard errors which allow the error term to be correlated over time for each administrative unit. In electronic supplementary material, we also present results from estimating the model parameters by allowing for time-invariant random effects that are specific to the administrative units, using generalized least squares. The estimated coefficients and their standard errors are appreciably the same between the baseline and the random effects specifications, which suggests that the conditioning variables in Z have absorbed the relevant dimensions of variation between administrative units, allowing us to identify the effect of smallpox experience.

We also consider a nonlinear specification. The specification in (2.1) assumes that the marginal effect of *h* on y is linear, albeit time dependent. However, our measures of vaccine refusal are in terms of proportions of infants and are thus bounded in the interval [0,1]. Observed outcomes are all well below the upper bound (table 1) but a few observed outcomes are at or near the lower bound. We thus also present results from a nonlinear specification using fractional regression analysis [51,52] that models the conditional mean of the dependent variable as a logistic function of the explanatory variables (in electronic supplementary material, we show further robustness to assuming a probit function for the mean). Denoting by *x* the vector containing the explanatory variables in (2.1) (including the constant term), and by β the associated vector of parameters, the conditional mean in the fractional regression context is given by

2.2 $$E(y|x)=\frac{\exp(x\beta )}{\left[1+\exp(x\beta )\right]}\;.$$

**Table 1.** Descriptive statistics of variables in our analysis across wards in Glasgow (descriptive statistics for vaccine refusal rate are across wards and years 1907–1913). Units are provided in brackets where appropriate. Details of the construction of variables and data sources are provided in the electronic supplementary material.

variable	minimum	maximum	mean	s.d.
vaccine refusal rate (proportion)	0	0.355	0.136	0.071
smallpox cases	7	617.2	109.3	148.6
smallpox case rate (per 1000 population)	0.129	6.605	1.533	1.589
smallpox deaths	0	68.56	12.44	16.06
smallpox death rate (per 1000 population)	0	0.728	0.171	0.171
population density (persons per hectare)	27.18	551.0	224.4	147.3
rooms per dwelling (ward average)	1.760	6.820	3.065	1.507
Irish born (per cent)	2.320	16.25	8.358	3.747

The parameters are estimated by quasi-maximum-likelihood (QMLE) with robust standard errors which, as in the case of the linear specification, allow for clustering of the error term by administrative unit.

### (c) Data

We use the municipal wards of Glasgow as the main unit of analysis. The ward was the principal administrative unit used by the Medical Officer of Health as the most appropriate geography to examine variation in health outcomes within the city of Glasgow [53] (for additional analysis of different geographies and the use of municipal wards by the Medical Officer of Health see electronic supplementary material, Section A). Additional ward-level data are also available for census years and allow us to capture relevant SC [53,54]. We summarize, in this section, the key variables we use in our analysis (see table 1 for descriptive statistics); further detail of variable construction is provided in electronic supplementary material, Section A.

At ward level, we define the vaccine refusal rate $y_{it}$ as the ratio computed as the number of children in ward *i* and year t (after 1907) whose parents registered COV of their child divided by the number of children born in that year in that ward. In Results, the smallpox experience variable $h_{i}$ is expressed as the average death rate across the two epidemics between 1900 and 1904, measured in deaths per thousand residents. In electronic supplementary material, Section A, we show that cases and deaths by ward were highly correlated between the two epidemics, which is why we average the death rate over the two consecutive epidemics. We also show that counts of deaths were highly correlated with the death rate across wards and that cases were highly correlated deaths (both for counts and rates). Our main findings are very similar if we use counts of cases or deaths, or the case rate, instead of the death rate, as our measure of h (electronic supplementary material, Section B). Information on the smallpox variables for the 1900 epidemic was recorded in the Medical Officer of Health reports by sanitary district and for the 1903 epidemic by ward; we thus convert the sanitary district variables into the relevant wards by using detailed maps [36] from the relevant time periods and information on building densities (see electronic supplementary material, Section A for a full description).

The SC included in Z consist of (i) rooms per dwelling; (ii) population density and (iii) per cent Irish born. We use ward-level data based on the 1911 census for the first two variables; information on the percentage of individuals born in Ireland is only available in the 1901 census. Our rationale for including these measures is as follows. Rooms per dwelling, measured as the average number of windowed rooms across dwellings in a ward, is used to approximate socioeconomic status in a ward. Socioeconomic status may have influenced the parental decision to vaccinate their children via its implications regarding the importance of cost considerations. As described earlier, both vaccination and submitting conscientious objection implied financial and time or other administrative costs. These costs included, for example, the costs of travel to the site of vaccination or the location of the solicitor, and additional travel or postal costs required to deliver the exemption certificate or proof of vaccination to the registrar. Even if it was financially less costly to choose one option relative to the other, the time and other administrative costs associated with that option might have affected the balance. How much the net financial or time cost mattered for a household probably depended on the socioeconomic status of the family. For example, for wealthier households, it is likely that differences in the financial implications of the two options had little bearing on the decision, whereas time cost differences might have been critical. We thus need to partial out possible effects of socioeconomic status on vaccine refusal that might have acted via these cost considerations. There could also be additional reasons, beyond cost considerations, for which socioeconomic status affected vaccination decisions. For example, historical analysis documents that, in England, the anti-vaccination movement in the nineteenth century was stronger among lower social classes [15,41]. Higher population densities may facilitate the spread of disease and thus imply a higher risk of contracting disease. Hence, to the extent that such risk was internalized, higher population density should, other things equal, tend to reduce vaccine refusal. However, higher population density in a ward was also associated with lower socioeconomic status (indeed, there is a negative correlation between rooms per dwelling and population density for wards in Glasgow; see electronic supplementary material, Section B), so that population density may pick up effects associated with socioeconomic status more generally. Regarding the variable per cent Irish born, our motivation is to acknowledge potentially different collective experiences of disease and related social norms for parents born outside of Glasgow, as well as the ability of new arrivals to navigate administrative procedures. Irish born individuals made up a relatively large share of the population in Glasgow, but one that was unevenly spread across wards, ranging between about 2.5% and 16% [53]. Vaccination rates were generally as high or higher in Ireland (over 90% in the 1870s) than in Scotland and there were relatively low levels of anti-vaccination sentiment, perhaps as the result of a more flexible and local approach [34]. Thus, if anything, Irish born individuals would be expected to be less likely than locally born parents to refuse vaccination of their children. At the same time, the negative correlation between rooms per dwelling and per cent Irish born for wards in Glasgow (electronic supplementary material, Section B) suggests that the effects of Irish immigrants may be entangled with those of socioeconomic status of the wards. Therefore, for both population density and per cent Irish born, negative effects on vaccine refusal are likely to be conditional on partialling out effects associated with socioeconomic status, captured here by rooms per dwelling.

We also present results using registration districts as the administrative unit of analysis. Registration districts were larger and more socioeconomically heterogeneous than wards (see electronic supplementary material, Section A). However, there is more detailed information on vaccination outcomes for registration districts, which allows us to establish the robustness of the main results. For registration districts, in addition to vaccine refusal data, Registrar General reports contain information on all possible outcomes relevant to vaccination for children born in a given year in the registration district; this allows us to trace cohorts born in each year between 1907 and 1913 in terms of the full range of vaccination outcomes in their first year of life (see electronic supplementary material, Section A for details). We thus calculate the proportion of children whose parents submitted a COV certificate in that year and registration district as a share of children who did not die before vaccination and denote the respective variable COV. We also construct a variable, denoted RU, calculated as the children ‘removed from the district or otherwise unaccounted for’ as a share of children who did not die before vaccination, and a third variable, denoted CR, defined as the sum of COV and RU. CR provides an alternative, broader, measure of vaccine hesitancy that includes both vaccination refusal (via the mechanism of formal conscientious objection) and potential informal evasion or laxity (via increases in the ‘otherwise unaccounted for’ component of the RU category after 1907; indeed, laxity was raised as a possible concern by the Medical Officer of Health [55]). For this analysis, the variable rooms per dwelling is calculated from data provided at the registration district level in the 1911 census data. Population density is calculated from population data in the 1911 census and the area calculated from maps of registration districts [36]. The smallpox experience variable *h* and the per cent of Irish-born residents (from 1901) are converted to registration districts from the relevant ward-level information using maps of Glasgow [36] and information on building density to distribute ward-level information across the relevant registration districts (see electronic supplementary material, Section A for details).

## 3. Results

**Figure 2.**
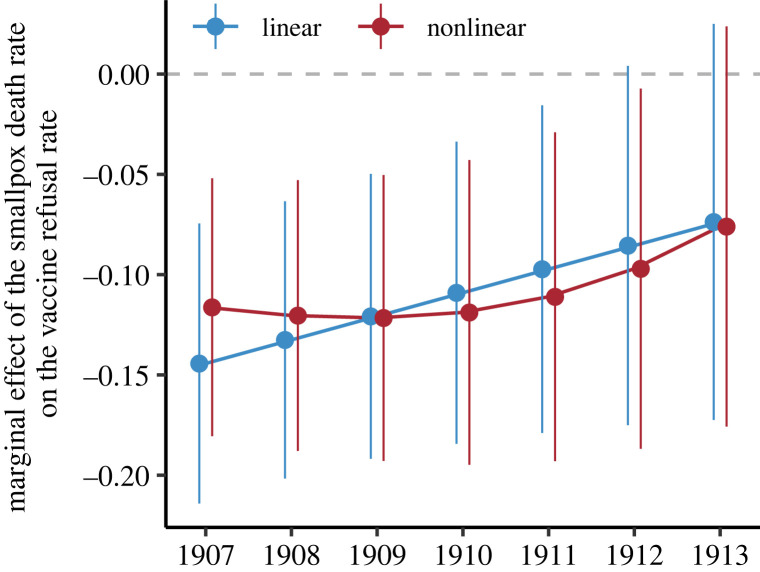
Estimated marginal effect of the smallpox death rate during the 1900 and 1903 epidemics on the vaccine refusal rate in each year, with 95% confidence intervals, for the linear and nonlinear models. The marginal effects are calculated as the effect of an increase of one unit in the smallpox death rate on the vaccine refusal rate for the year shown on the *x*-axis.

Table 2 shows the estimates from the model specifications in (2.1) and (2.2) using municipal ward data. Figure 2 shows the corresponding estimated marginal effects of $h_{i}$ and their 95% confidence intervals, over the 7 years.

**Table 2.** Coefficient estimates of models of vaccine refusal rate in wards of Glasgow, 1907–1913. Least-squares estimation (for linear specification in equation (2.1)) and fractional regression (QMLE) estimation (for nonlinear specification in equation (2.2)). Robust s.e. (ward-level clustered) are shown in parentheses under the estimated coefficients. The dependent variable in both models is the vaccine refusal rate. Number of observations: 175 (25 wards); ***p < 0.001; **p < 0.01; *p < 0.05; *p*-values are based on two-sided *t*-tests of statistical significance of the coefficient.

	smallpox death rate (linear)	smallpox death rate (nonlinear)
(intercept)	2.68 × 10^−01^*** (4.35 × 10^−02^)	−7.18 × 10^−01^ (3.87 × 10^−01^)
smallpox	−1.56 × 10^−01^*** (3.72 × 10^−02^)	−1.88 × 10^0^*** (4.93 × 10^−01^)
smallpox × year	1.18 × 10^−02^ (6.87 × 10^−03^)	2.03 × 10^−01^*** (5.36 × 10^−02^)
year	2.07 × 10^−02^*** (3.11 × 10^−03^)	1.69 × 10^−01^*** (2.23 × 10^−02^)
population density	−2.17 × 10^−04^*** (4.41 × 10^−05^)	−1.87 × 10^−03^*** (3.53 × 10^−04^)
rooms per dwelling	−3.01 × 10^−02^*** (7.46 × 10^−03^)	−2.63 × 10^−01^*** (6.49 × 10^−02^)
per cent Irish born	−6.67 × 10^−03^** (2.05 × 10^−03^)	−5.88 × 10^−02^** (1.92 × 10^−02^)
*R*^2^	0.64	–

The main conclusion from the results in table 2 and figure 2 is that a lower smallpox death rate during the epidemics between 1900 and 1904 was associated with higher rates of vaccine refusal after 1907, an association that was stronger in the years closer to the 1900–1904 smallpox epidemics. Indeed, the marginal effects in figure 2 are negative, being larger and significantly negative at the 95% level for the first 5‐6 years. Wald tests shown in the electronic supplementary material, Section B, also confirm that the model specifications including the smallpox variables are preferred over specifications without them. In figure 2, the nonlinear models reveal a small nonlinearity in the marginal effect that increases the persistence of the effect of smallpox experience on vaccine refusal; otherwise, the results are quantitatively similar to those of the linear models.

Regarding the socioeconomic variables, the estimates suggest a negative relationship between vaccine refusal and population density, rooms per dwelling and per cent Irish born. Given the discussion in Data, the negative effect of the per cent Irish born likely reflects a more favourable view of vaccination among Irish immigrants, while the negative effect of population density is consistent with an increased acceptance of vaccination when disease risk is higher (in more crowded neighbourhoods). The negative estimated coefficients of these variables (table 2) are statistically significant, a result of accounting for the effects of socioeconomic status via rooms per dwelling. Indeed, as shown in table S8 in the electronic supplementary material, when rooms per dwelling is omitted, implying that population density and per cent Irish born also pick up effects of socioeconomic status in the model, then the estimated coefficients of these two variables are smaller in absolute value and no longer statistically significant. These considerations imply that the effect of socioeconomic status on vaccine refusal was negative (if it were positive, in its absence, the estimated coefficients for population density and per cent Irish born would be even more negative and significant), which is indeed confirmed by the estimate of rooms per dwelling in table 2. The finding that socioeconomic status was negatively associated with vaccine refusal might imply that the total financial cost of the process of vaccination was higher than that of conscientious objection such that poorer households, for whom this cost should be more important, would tend to refuse vaccination; it could also imply that conscientious objection carried a non-trivial time cost, which should have been more important for the wealthier households; or it could suggest a more general positive association between socioeconomic status and stronger anti-vaccination sentiment in Glasgow, as is documented in England.

Results summarized in electronic supplementary material, Section B show that the baseline results are similar in magnitude and significance when parameters are estimated by modelling ward-level unobservable characteristics as random effects in the linear model, by allowing for a full set of year dummy variables (instead of the linear trend) and by assuming a probit functional form specification for the nonlinear model. They also show that if we add to the model specification a variable capturing the distance to the Belvidere smallpox hospital, its effect is not significant and does not change the significance of the remaining estimated parameters or the main patterns discussed above (for a detailed discussion, see electronic supplementary material, Section B), suggesting that relevant effects from proximity to the hospital are already accounted for by the independent variables included in the specifications in (2.1) and (2.2).

**Figure 3.**
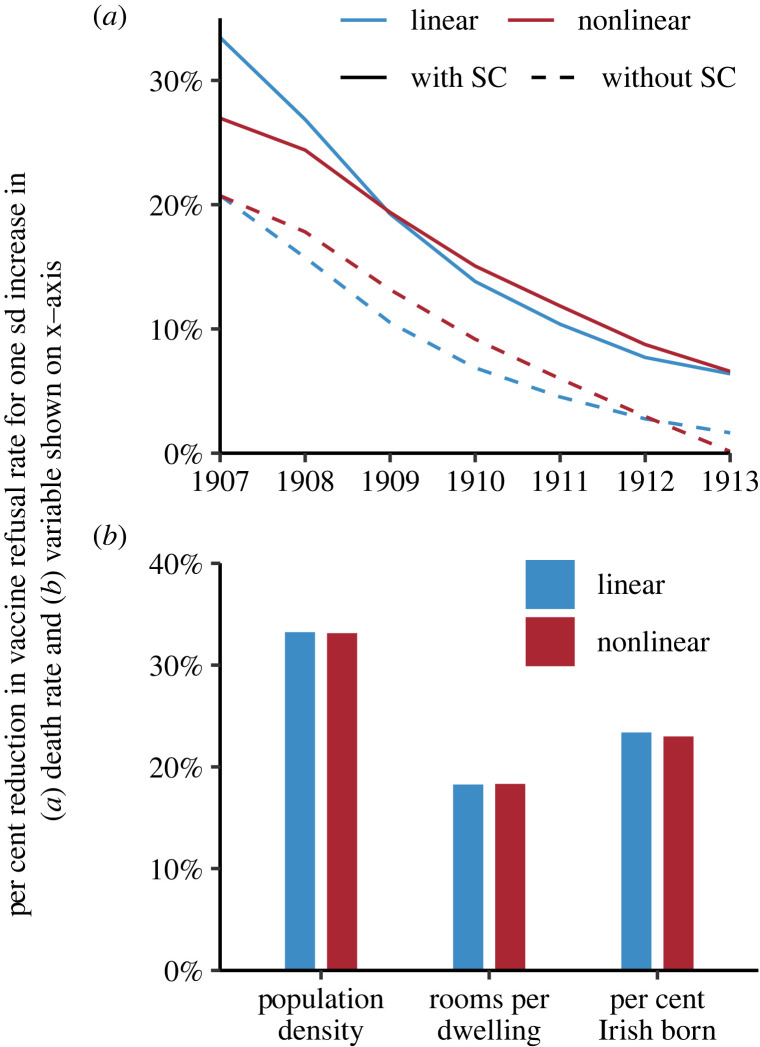
(*a*) Per cent reduction in vaccine refusal rate given a one s.d. increase in the smallpox death rate during the 1900 and 1903 epidemics for the linear and nonlinear models. The effects are expressed as the per cent reduction in vaccine refusal relative to the average vaccine refusal rate in the corresponding year. The panel also includes results for a specification without variables relating to SC (coefficient estimates for these models are provided in electronic supplementary material, Section B). (*b*) Per cent reduction in vaccine refusal rate given a one s.d. increase in the independent variable shown on the *x*-axis, for linear and nonlinear models. The effects are expressed as per cent reduction in vaccine refusal relative to the average vaccine refusal rate across all years.

To further illustrate the importance of the estimated marginal effects of prior disease experience, and to contextualize them relative to the marginal effects of the other variables, we present, in figure 3, the proportional reduction in vaccine refusal rate that would result, other things equal, from a one s.d. increase in the smallpox death rate (figure 3*a*) and the socioeconomic variables (figure 3*b*). The main conclusion we draw from figure 3 is that this standardized effect of prior disease experience on vaccine refusal was substantial in magnitude and comparable to the effect of the SC during the first 3–4 years. Comparing the model specifications with and without socioeconomic variables, the model including the socioeconomic variables is supported by Wald tests relative to the model excluding them (see electronic supplementary material, Section B). In figure 3, we see that the standardized smallpox experience effects are about one third smaller in the specification where we do not include socioeconomic conditions; this difference in the size of the effects reflects the bias due to confounding associated with SC. We thus conclude that including the SC is important to estimate the association between disease experience and vaccine refusal. Given the high correlation between disease experience variables, the equivalent results to those shown in figure 3 are very similar if, instead of the smallpox death rates during the epidemics of 1900–1904, we use as a measure for *h* smallpox deaths, cases or case rates (see electronic supplementary material, Section B).

### (a) Extensions

**Figure 4.**
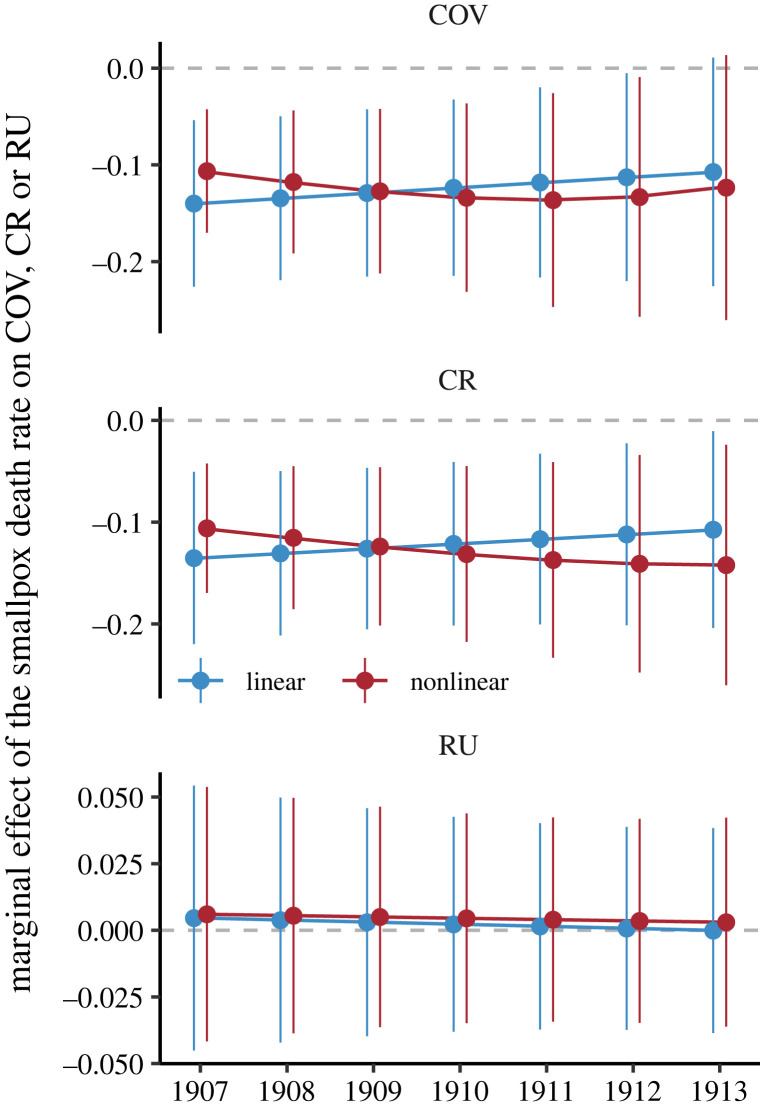
Estimated marginal effect of the smallpox death rate during the 1900 and 1903 epidemics on three vaccination outcomes constructed using registration district data in each year, with 95% confidence intervals, for the linear and nonlinear models. The vaccination outcomes are COV; COV plus removed from district or otherwise unaccounted for (CR); removed from district or otherwise unaccounted for (RU). The marginal effects are calculated as the effect of a change of one unit in the smallpox death rate on the relevant vaccination outcome for the cohort born in the year shown on the *x*-axis.

The main results are robust to estimating models (2.1) and (2.2) using registration districts as the administrative unit according to which the relevant variables are measured. Figure 4 shows the marginal effects of death rates on COV, CR and RU (see Data for the construction of these variables) under the different model specifications, confirming that prior smallpox experience had a significant negative association with both COV and CR (see electronic supplementary material, Section C for full tables of estimates for analysis by registration district). The results regarding CR, in particular, suggest that the negative association is robust to allowing for some level of vaccine hesitancy to be expressed via an increase in laxity towards vaccination obligations after 1907, as captured by any contribution to the increase in RU of the ‘otherwise unaccounted for’ component. On the other hand, we do not find a significant effect of prior smallpox experience on RU alone. Focusing on the marginal effects of COV in figure 4 and comparing these with the marginal effects using ward-level data in figure 2, we see that the effects of smallpox experience appear to reduce more slowly in the analysis by registration district. A possible explanation is that households moved between registration districts less frequently than between wards because registration districts were larger (see Methods and electronic supplementary material, Section A).

Finally, we undertake additional experiments in which smallpox experience in the baseline specifications (2.1) and (2.2) is replaced by mortality due to measles or to scarlet fever (each showing certain similarities to smallpox; see electronic supplementary material, Section C) over the same years as the smallpox measures. Variation in mortality between wards in these two diseases is comparable to that of the SC (as measured by the coefficient of variation; see also table 1), although lower than variation in smallpox mortality. The results, in electronic supplementary material, Section C, show that the effect of measles or scarlet fever on smallpox vaccination refusal is not significant, reaffirming the importance of smallpox experience specifically.

## 4. Discussion and conclusion

Our findings contribute to the substantial and growing literature on factors associated with vaccination decisions by revealing that lower local disease incidence and mortality during earlier epidemics can contribute to higher rates of subsequent vaccine refusal. In Glasgow, at the start of the twentieth century, our findings indicate that this effect acted in addition to those of SC and had a similar magnitude to these, at least for a few years following the relevant disease experience. To the extent that local area disease experience also reflects the probability of knowing someone who contracted or died from a disease, our results suggest that personal experience is a contributing factor to decisions to vaccinate, at least in situations where individuals can legally choose whether or not to do so. Because our results are obtained for a period during which disease outbreaks were still relatively common, they imply that complacency is not a phenomenon only of recent decades, resulting from the generally low levels of disease risk. Instead, they suggest that complacency also influenced vaccination decisions in periods of higher infectious disease risk, responding to local prior experience of the relevant disease.

Possible extensions to this work would include investigating the importance of the strength and form of social networks within geographical areas, as well as the kinds of personal experience that are most important for driving vaccination decisions (e.g. friends versus relatives or whether the sick individual is a child or an adult), and whether the vaccines in question are routinely given to the individual making the decision or to their children.

To consider the relevance to other periods and geographical areas of our results regarding the importance of prior local disease experience for vaccination decisions, it is important to understand and determine the relevant spatial area, taking into account the strength and form of social interactions within that area. In particular, increased travel and mobility in modern societies means that the relevant geographical areas are likely to be larger than in the past. Moreover, social interactions now depend less on physical distance, suggesting that the effect of geography should be complemented by that of social networks not defined by physical space. Indeed, the effect of local area variation needs to be studied jointly with the strength of the social interactions that the local areas imply. Nonetheless, there are still places—when defined at the appropriate scale—where social interactions at a local scale remain very important, for example for geographical, economic or cultural reasons. In these areas, a stronger prior disease experience should, other things equal, imply lower vaccination refusal. The implication is that conditional on identifying geographical areas with strong local social interactions, vaccine policy may be more effective if it takes into account prior local disease experience. Indeed, spatially localized pockets of unvaccinated individuals make an important contribution to disease re-emergence [17,19,23,56], and therefore identifying where these might arise is critical to the design of vaccine roll-out strategies.

## Data Availability

The original data used in this study are available in the public domain from Wellcome Library online, Glasgow City Archives or University of Glasgow library and can be accessed using the information provided in the manuscript, electronic supplementary material and associated references. Data and R code required for all the analyses can be accessed Dryad Digital Repository [57]. Additional information about the data is provided in the electronic supplementary material [58].
